# Physical Fitness and Upper Limb Asymmetry in Young Padel Players: Differences between Genders and Categories

**DOI:** 10.3390/ijerph19116461

**Published:** 2022-05-26

**Authors:** Francisco Pradas, Víctor Toro-Román, Miguel Ángel Ortega-Zayas, Duber Mary Montoya-Suárez, Bernardino Javier Sánchez-Alcaraz, Diego Muñoz

**Affiliations:** 1 ENFYRED Research Group, Faculty of Health and Sports Sciences, University of Zaragoza, 22001 Huesca, Spain; franprad@unizar.es (F.P.); maortega@unizar.es (M.Á.O.-Z.); montoyasuarez6@gmail.com (D.M.M.-S.); 2 School of Sport Sciences, University of Extremadura, 10003 Cáceres, Spain; diegomun@unex.es; 3 Department of Physical Activity and Sport, Faculty of Sport Sciences, University of Murcia, 30700 Murcia, Spain; bjavier.sanchez@um.es

**Keywords:** racket sports, fitness, under-14, under-16, male, female

## Abstract

This study aimed to assess the physical fitness and upper body asymmetries of young padel players aged between 13 and 16 years and to determine the possible differences between genders and categories. A total of 60 padel players were divided into four groups: under-14 male (*n* = 15; age: 13.75 ± 0.45 years; height: 1.64 ± 0.07 m; weight: 54.7 ± 8.3 kg), under-14 female (*n* = 15; age: 13.75 ± 0.44 years; height: 1.60 ± 0.05 m; weight: 51.5 ± 6.0 kg), under-16 male (*n* = 15; age: 15.44 ± 0.51 years; height:1.71 ± 0.04 m; weight: 63.88 ± 6.2 kg) and under-16 female (*n* = 15; age:15.46 ± 0.52 years; height:1.63 ± 0.05 m; weight: 55.08 ± 3.6 kg). Handgrip strength, ischiosural flexibility, gestural speed of the dominant arm, vertical jump, cardiorespiratory capacity, lateral movement, lateral acceleration and reaction time were measured. Male players showed better results in manual grip strength, vertical jump power, cardiorespiratory capacity and lateral movement (*p* < 0.05). Moreover, males presented a higher percentage of asymmetry in upper limb strength. Female players showed better reaction time and greater flexibility (*p* < 0.05). Regarding the differences between categories, the under-16 players showed greater flexibility, gestural speed, vertical jump power, cardiorespiratory capacity and lateral movement compared to the under-14 players. These results can be used as reference values for coaches/physical trainers of younger categories to improve health control and physical performance planning.

## 1. Introduction

Padel is a racket sport that has become very popular worldwide, increasing its practice in the last decade among people of all ages, genders and fitness levels [1]. Interest in the practice of this sport has grown mainly due to psychosocial aspects [2,3] and the possible benefits for physical fitness and body composition [4]. In the last 5 years, the number of federative licenses has increased from 58,324 in 2016 to 96,872 in 2021, with 65% in the male categories and 35% in the female categories [5,6]. In addition, 13.2% correspond to players under 19 years, a percentage that increases annually.

Padel is an intermittent sport played in pairs on a 20 × 10 m court surrounded by glass walls and a metal fence on which the balls can bounce [7]. It can be considered a predominantly aerobic sport with short periods of high and very high-intensity action in which the phosphagen system (ATP-PCr) prevails [8]. Frequent actions occur during a padel match (0.7–1.5 per second) [9], followed by rest periods as defined in the rules [10]. Therefore, players must perform fast movements and constant changes of direction for which optimal levels of strength, agility and speed are required [1].

In recent years there has been increasing interest in studying the sport of padel by analysing adult players of different levels and especially professional players [11,12]. The found published research has focused mainly on investigating other game variables, such as the temporal structure [13,14,15,16] and the technical or tactical actions performed during the matches [17,18,19,20] as well as analysing the metabolic and physiological response that occurs during the competition [1,8,21]. From a fitness point of view, a recent study compares the existing differences in physical fitness parameters between genders in professional players. It was observed that male players presented better results in maximal strength, explosive strength and oxygen consumption tests [9]. However, the literature concerning the analysis of physical fitness in players at the formative stages is scarce. A study in which physical fitness was evaluated in young padel players differentiating between genders and experience [22] reported that experience could influence lateral throwing strength with a medicine ball and was related to improvements in strength in lateral actions characteristic of racket sports. In another similar study, conducted with a smaller sample of players aged 11–16 years, it was concluded that males were faster, more agile and stronger than females [23]. 

Several factors, such as anthropometry and physical fitness, have been considered predictors of sports performance and possible selection parameters for young athletes [24,25]. The importance of each of the factors that determine sports practice and performance depends on the level of the athletes, their gender, the moment in the training process or the context of practice [26]. In addition, monitoring the evolution of these factors makes it possible to establish correct training planning, which should consist of periodic evaluations and follow-ups of the players. The performance of physical tests by padel players could improve their training processes, identify future talents and detect possible injuries [9,22]. In addition, during puberty, changes in anthropometric and physical fitness parameters become obvious, not only in subjects of the same gender but also in individuals of opposite genders [27], which would emphasise the importance of following the evolution of these parameters at these ages. 

Moreover, it should be noted that padel is an asymmetric sport, where the volume of repetitions of the dominant limb can create both anatomical and physical and physiological differences (muscle decompensations) that lead to an increase in the risk of injury and long-term health problems. Previous studies in sports such as tennis have observed differences in both circumference and muscle volume in young players between dominant and non-dominant sides [28] and differences in the range of motion between the two upper extremities [29]. 

Due to the maturational changes and gender distinctions discussed above, we hypothesize that there will be differences in physical fitness parameters. Therefore, the objectives of the present study were to assess the physical fitness values and upper body asymmetries in young padel players between 13 and 16 years, both male and female, and to determine the possible differences between genders and categories.

## 2. Materials and Methods

### 2.1. Participants

The present study was conducted on a target population of 131 federated players in the under-14 and under-16 categories in the community of Aragon (Spain), obtaining a minimum sample size of 60 players (confidence level = 95%; margin of error = 10%). A total of 60 padel players were divided into four groups: under-14 male (*n* = 15), under-14 female (*n* = 15), under-16 male (*n* = 15) and under-16 female (*n* = 15) (Table 1). All the participants had to meet the following inclusion criteria: (i) to be between 13 and 16 years old; (ii) not to have any injury or illness during the investigation or at least six months before the study; (iii) to have at least two years of padel experience; (iv) to practise only the sport of padel; (v) to participate in federated competitions. All the participants had a minimum of three training sessions per week.

The subjects and their parents or guardians were informed of the aim of the study and signed a consent form for participation in it. The protocol was reviewed and approved by the Clinical Research Ethics Committee of the Department of Health and Consumption of the Government of Aragon (Spain) (21/2012), following the guidelines of the Helsinki Ethical Declaration, updated at the World Medical Assembly in Fortaleza (2013) for research on humans. Each participant was assigned a code to maintain anonymity.

### 2.2. Procedures

Before the assessments, the players underwent a familiarisation session with all the assessment tests. Participants and their coaches were requested to abstain from intense activities for 48 h before the assessments. The assessments were always performed at the same time (9:30 a.m.) and under the same environmental conditions (~20 °C, ~60% humidity). Before the familiarisation session and assessments, a standardised 15 min warm-up based on general mobility and low-intensity continuous running was performed. Participants rested 3–5 min between each test.

### 2.3. Anthropometric Measures

Body weight and height were obtained using a scale (Seca 769, Seca, Hamburg, Germany) and tape (Seca 220, Seca, Hamburg, Germany) with an accuracy of ±0.001 kg and ±0.001 m in nude, barefoot conditions. The body mass index (BMI) values were obtained from the above parameters.

### 2.4. Hand Grip Strength Measurement

Participants performed two maximal voluntary contractions with the arm fully extended on the vertical axis and without touching the body. Dominant and non-dominant hands were evaluated. The dynamometer grip was adapted to the participants’ hands [30]. Manual grip strength was measured with a Takei 5101 dynamometer (Takei Instruments Ltd., Tokyo, Japan) with a range between 0 and 100 kg, with increments of 0.5 kg and an accuracy of ±2 kg. Participants made two attempts of 3 s duration, and the highest value was selected for analysis. Rest time between each effort was 1 min. Asymmetry was obtained from the differences between the dominant and non-dominant arm.

### 2.5. Tapping Test

The plate tapping test was used to assess the gestural speed of the dominant arm. For the test, the table and the chair were adjusted to a comfortable height for the subject. The table had two drawn circles of 20 cm in diameter 60 cm apart with a 30 × 20 cm plate between the two circles. The performer positioned himself in front of the table with his feet slightly apart, placing his non-dominant hand on the rectangle and the other hand on one of the circles. After the start signal, the subject alternately touched the two circles a total of 25 times each with the dominant hand at the highest possible speed. The test ended at the 50th contact. Two test subjects monitored the time and number of contacts. A Casio HS-80TW-1EF stopwatch (Tokyo; Japan) was used to measure the total time.

### 2.6. Flexibility Measurement

The sit and reach test was used to measure the range of motion of the lower back and hamstring muscles [31]. From a seated position on the floor with legs fully extended, participants pushed the measuring scale as far as possible without bending their knees, placing one hand on top of the other or side by side with palms down. The evaluator helped to keep the knees extended and downward. Participants held the final position for one to two seconds while the distance in cm was recorded. The best of the two repetitions was chosen for analysis.

### 2.7. Vertical Jump Measurement

Lower limb explosive strength was assessed using the squat jump (SJ), countermovement jump (CMJ) and Abalakov jump (ABK) tests. These tests were selected due to their high reliability [32]. A jump mat system (Newtest Powertimer^®^, Oulu, Finland) was used to measure jump height. Two attempts were made for all jumps with a 60 s rest between jumps. The best jump was selected for further analysis. The protocols for each jump test conducted followed the guidelines proposed by Bosco et al. [33]. 

To perform the SJ, participants started in a semi-flexed position with knees at 90° and hands on hips. A goniometer was used to verify the knee angle. Participants were required to remain in this squat position for 3 s before performing the jump [34]. For CMJ, subjects started from an upright position with their hands on their hips. Then, they performed a rapid knee and hip flexion movement in a single sequence, followed immediately by a rapid vertical movement to jump as high as possible. Finally, during the ABK test, participants initiated the upright position and, in a single action, had to perform a knee and hip flexion movement followed by a quick extension movement with the help of the arms to propel themselves. The SJ and CMJ jump power was obtained using the equation proposed by Sayers et al. [35].

### 2.8. Course Navette Test

Cardiorespiratory capacity was assessed using the course navette test [36]. This test consisted of out-and-back runs of 20 m in several stages [37]. A photocell system (Newtest Powertimer^®^, Oulu, Finland) was used to monitor the run. From one point to another, sound signals were emitted from a pre-recorded tape that increased 0.5 km·h^−1^ every minute from an initial speed of 8.5 km·h^−1^. When the subject could no longer maintain the pace, the last announced stage number was used to estimate maximal oxygen consumption (VO_2max_) using the formula specified in the literature [37].

### 2.9. Accelerations, Lateral Displacements and Reaction Time Measurement

**Figure 1:**
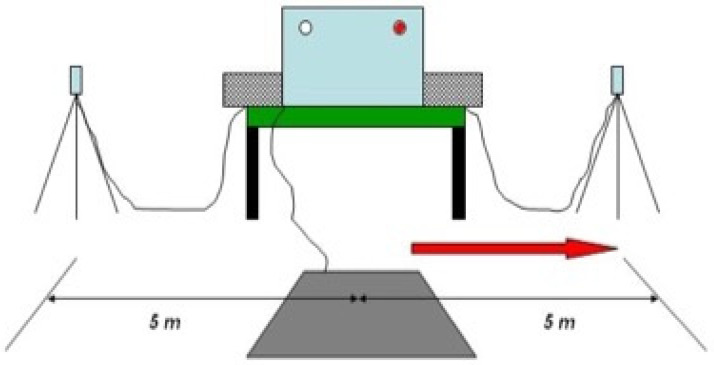
Take-Off reaction test.

Subjects performed the Take-Off Reaction Test (Newtest Powertimer^®^, Oulu, Finland) (Figure 1). Participants initiated the action, left or right, from the padel rest position, after reacting to light (left or right) emitted randomly by a device. Subjects left the contact mat and made a lateral run to the left or right photocells (placed 5 m from the mat). Subjects completed 12 attempts (six to the left and six to the right) and recorded the best result. All attempts were performed consecutively with a brief 30 s rest period. The protocol was similar to that employed by Castellar et al. [38]. 

The parameters measured were (a) reaction time: time elapsed between turning on the lights and the moment when the subjects took their foot off the mat; (b) acceleration: change in velocity from the start of lateral movement until the photocell barrier was passed; (c) lateral movement: time elapsed between turning on the lights and the moment when the subjects passed the photocell barrier. Anticipation was considered when the players reached a reaction capacity of <150 ms. The photocells were placed approximately at hip level to avoid any possible interference of the upper limbs.

### 2.10. Statistical Analysis

Data are expressed as means and standard deviation. Statistical analysis was performed with version 22.0 of IBM^®^ SPSS^®^ Statistics (IBM Corp., Armonk, NY, USA). The Shapiro Wilks test was applied to study the normality of the variables. Likewise, Levene’s test was used to determine the homogeneity of variances. A two-way ANOVA (group x gender) was used to show the differences between the variables studied. The effect size (ES) was calculated using partial eta-squared with values interpreted as low effect (0.01–0.06), moderate effect (0.06–0.14) and high effect (>0.14) [39]. A value of *p* < 0.05 was considered statistically significant.

## 3. Results

The results obtained in the present investigation are shown below. Table 1 shows the characteristics of the sample according to the different groups. Differences between categories were observed in all parameters (*p* < 0.01). Regarding the differences between genders, there were differences in height and weight (*p* < 0.01). 

Table 2 shows the results obtained in hand grip strength, the tapping test and flexibility. Regarding category, there were differences in the tapping test and flexibility (*p* < 0.05). Concerning gender differences, significant differences were observed in dominant and non-dominant hand grip strength, asymmetry and flexibility (*p* < 0.001).

Table 3 shows the results obtained in the vertical jump tests. There were significant differences in all the parameters analysed when comparing genders (*p* < 0.01). Regarding the differences between categories, there were substantial differences in absolute power and relation to weight in the SJ and CMJ tests (*p* < 0.001).

Table 4 shows the results obtained in the course navette test. There were differences concerning gender in all the parameters analysed (*p* < 0.001). Regarding differences between categories, there were differences in absolute VO_2max_ (*p* < 0.05).

Finally, Table 5 shows the values obtained in the lateral movement, acceleration and reaction time tests on the dominant and non-dominant side. There were significant differences concerning category in the lateral movement time on the dominant side (*p* < 0.05). On the other hand, regarding differences between genders, there were differences in lateral movement (dominant and non-dominant), acceleration on the dominant side and reaction time on the non-dominant side (*p* < 0.05).

## 4. Discussion

Research on the assessment of physical fitness in padel players has been increasing in recent years [9,40] due to its interest and importance for optimal performance in this sport. However, in youth categories, the literature is scarcer [22]. The present study provides new data concerning physical fitness in young padel players and its evolution according to category (under-14 and under-16). With respect to the results obtained, males and under-16 players generally obtained better results in all the parameters analysed, possibly due to hormonal differences between gender and age [41]. In addition, greater asymmetries were observed in the upper limb in males. The research adds information on the results for hand grip strength, explosive strength of the lower extremities and gestural speed and the speed, acceleration and time of lateral movement.

### 4.1. Vertical Jump 

Studies that have analysed performance factors in padel conclude that winning pairs are those that manage to stay longer in the net area [7,20,42], performing more attacking actions than losing pairs [43,44], specifically smash and volley actions [45]. These data indicate the relevance of all manifestations of strength, both gripping and jumping. In this respect, the results obtained in jumping tests (SJ and CMJ) are lower compared to other racket sports [46,47,48]. The lower intensity of the game, of actions involving jumping and the type of movement required in padel could explain these results [22]. Regarding the game categories, previous studies in tennis players observed similar differences between different categories [49,50]. In padel, similar differences between genders have been observed [9,22]. Sánchez-Alcaraz et al., found that male players could perform finishing shots from distances farther from the net than female players [45], which could justify the better results obtained in this type of test. The fact that female players perform finishing shots close to the net could be related to better gestural speed since in this position, close to the net, it is very important to move the padel from the forehand to backhand position very quickly due to the high speeds at which the ball arrives at the height of the net.

### 4.2. Grip Strength and Asymmetry 

The gender differences in hand grip strength found in the present study are related to what has been observed in other investigations and also in padel [9,21]. The measurement of hand grip strength is widely used due to its simplicity, low cost and the reproducibility of the technique [49]. In addition, hand grip strength seems to be a good indicator of service speed in other racket sports [50], which could be related in turn with the speed of a padel player’s smash, as observed by Prieto-Bermejo and Renes-López, where higher-level players recorded higher smash speeds compared to players of a lower level of play [51]. Therefore, it is essential to monitor this parameter in young players, where many actions require high grip strength values, such as smashes or volleys. The gender differences in hand grip strength observed in the present study could be due to differences in muscle mass. It is well known that males tend to have more lean mass than females, thus influencing strength levels [52]. During puberty, there is a significant increase in hormones, such as growth hormone, insulin-like growth factor-1 and gender steroids increasing bone and muscle mass which could translate to higher strength levels [24,53].

Racket sports can cause chronic body asymmetries due to their unilateral nature [36,52,54]. In tennis, asymmetries start to be significant from prepubertal ages, being greater in males compared to females, as in the present study [52,55]. The asymmetries could be the result of the mechanical load generated in the dominant arm due to frequent muscle contractions, torsional forces and racket vibrations during the execution of the different strokes [28]. The differences could be related to differences in training intensity as players tend to train with higher intensity and reach higher ball speeds, generating greater mechanical load on the dominant upper extremity [56].

### 4.3. Flexibility 

Regarding posterior flexibility, previous research observed similar differences between genders in table tennis players [36], in professional padel players [9] and in tennis players of similar ages [57]. Women players present greater flexibility compared to men, possibly due to the anatomical design of the bony structures of the hips and pelvis (pelvic retroversion) and hormonal regulation (oestrogens) that generates less stiffness [58].

### 4.4. Cardiorespiratory Fitness

Concerning cardiorespiratory fitness, previous studies also observed gender differences in padel [9,21] and other racket sports [59,60]. A high cardiorespiratory capacity and muscular endurance can help players delay the onset of fatigue and aid in recovery, representing a distinguishing factor for the best racket sport players [15]. Different research has observed that level and gender influence the external load of padel matches [61,62]. Higher-level players tend to play points and matches of longer duration than lower-level players.

### 4.5. Lateral Movement, Acceleration and Reaction Time 

Regarding movement speed, acceleration speed and lateral reaction time, other authors observed differences between categories and genders using a similar test in table tennis players [38,63]. The observed gender differences could be related to the characteristic playing style of each gender [64]. Male players tend to play faster games with shorter points requiring greater travel speed and accelerations, in contrast to the type of play of female players, who show a slower and more conservative game with longer rallies and a greater use of lobs [65,66,67].

### 4.6. Limitations

The present study presents a series of limitations that should be taken into account when interpreting the results: (i) padel-specific tests have not been included [68]; (ii) it would be of great interest to know the state of biological maturation in order to establish relationships between this and the results of the different tests; (iii) the results presented have validity within the study population and (iv) the analysis has not been carried out considering the game side. More studies are needed to confirm the differences. In addition, obtaining other anthropometric parameters, such as muscle percentage or arm and thigh muscle area, may affect the results, since there seems to be a direct relationship between muscle areas and explosive strength activities (jumping and throwing).

## 5. Conclusions

According to the results, young male padel players showed better results in hand grip strength, vertical jumping power, cardiorespiratory capacity and lateral movement, while young female players showed better reaction time and greater flexibility. The under-16 players showed better posterior flexibility, gestural speed, vertical jumping power, cardiorespiratory capacity and lateral movement than the under-14 players.

Female players have lower upper limb asymmetry compared to male players in both categories.

Due to the level of the players and the scarce information on the assessment of physical fitness in this group, these results could be used as a reference by padel coaches/physical trainers, taking into account the maturational stage of the players.

## Figures and Tables

**Table 1:** Participants’ characteristics.

		Under-14	Under-16	Group Effect (*p* Value)	Gender Effect (*p* Value)	Group x Gender (*p* Value)
Age (years)	Male	13.75 (0.45)	15.44 (0.51)	<0.001	0.926	0.926
	Female	13.75 (0.44)	15.46 (0.52)			
Height (m)	Male	1.64 (0.07)	1.71 (0.04)	0.002	<0.001	0.242
	Female	1.60 (0.05)	1.63 (0.04)			
Weight (kg)	Male	54.74 (8.33)	63.88 (6.21)	<0.001	0.001	0.100
	Female	51.48 (6.00)	55.08 (3.62)			
BMI	Male	20.10 (1.71)	21.75 (1.78)	<0.001	0.152	0.228
	Female	20.01 (1.41)	20.64 (1.18)			
Experience (years)	Male	3.33 (0.65)	4.31 (0.60)	<0.001	0.812	0.482
	Female	3.18 (0.54)	4.38 (0.50)			

BMI: body mass index.

**Table 2:** Results obtained in hand grip, tapping and flexibility tests.

		Under14	Under16	Group Effect (*p* Value)	ES	Gender Effect (*p* Value)	ES	Group x Gender (*p* Value)	ES
Hand grip strength dominant (kg)	Male	35.40 (7.28)	35.03 (5.58)	0.693	0.003	<0.001	0.402	0.504	0.008
	Female	26.31 (3.93)	27.77 (3.05)						
Hand grip strength non-dominant (kg)	Male	30.52 (8.13)	30.59 (4.29)	0.357	0.016	<0.001	0.389	0.384	0.014
	Female	21.75 (3.41)	24.12 (2.92)						
Asymmetry (%)	Male	11.31 (5.09)	10.86 (2.85)	0.766	0.002	<0.001	0.406	0.393	0.014
	Female	5.82 (1.65)	6.75 (1.42)						
Tapping test (s)	Male	10.91 (1.09)	10.11 (1.23)	0.010	0.118	0.158	0.036	0.774	0.002
	Female	10.45 (0.97)	9.81 (0.56)						
Flexibility (cm)	Male	18.91 (6.38)	25.46 (8.94)	0.019	0.099	<0.001	0.262	0.394	0.014
	Female	29.31 (6.24)	32.42 (7.85)						

ES: effect size.

**Table 3:** Values obtained in the vertical jump.

		Under14	Under16	Group Effect (*p* Value)	ES	Gender Effect (*p* Value)	ES	Group x Gender (*p* Value)	ES
SJ (cm)	Male	22.09 (5.12)	25.53 (3.85)	0.081	0.056	0.008	0.125	0.091	0.060
	Female	21.22 (2.44)	21.16 (2.41)						
SJ (W)	Male	1765 (414)	2388 (397)	<0.001	0.259	<0.001	0.299	0.131	0.033
	Female	1565 (277)	1724 (246)						
SJ (W·Kg^−1^)	Male	32.19 (5.72)	37.26 (4.07)	0.007	0.128	<0.001	0.204	0.067	0.051
	Female	30.30 (3.10)	31.22 (2.92)						
CMJ (cm)	Male	25.68 (4.75)	27.72 (3.67)	0.391	0.014	0.001	0.183	0.190	0.032
	Female	23.70 (2.52)	23.27 (2.85)						
CMJ (W)	Male	2002 (398)	2555 (382)	<0.001	0.225	<0.001	0.331	0.086	0.037
	Female	1741 (289)	1894 (263)						
CMJ (W·Kg^−1^)	Male	36.52 (4.33)	39.89 (3.34)	0.031	0.085	<0.001	0.293	0.130	0.043
	Female	33.69 (2.80)	34.30 (2.95)						
ABK (cm)	Male	29.39 (5.72)	30.40 (3.96)	0.618	0.005	0.001	0.183	0.655	0.004
	Female	26.21 (2.87)	26.26 (3.10)						

SJ: squat jump; CMJ: countermovement jump; ABK; Abalakov jump; ES: effect size.

**Table 4:** Results obtained during the course navette test.

		Under14	Under16	Group Effect (*p* Value)	ES	Gender Effect (*p* Value)	ES	Group x Gender (*p* Value)	ES
Stage (n°)	Male	6.75 (1.61)	7.12 (0.80)	0.213	0.029	<0.001	0.417	0.851	0.001
	Female	4.53 (1.65)	5.03 (0.96)						
Distance (m)	Male	1129 (322)	1203 (160)	0.249	0.025	<0.001	0.405	0.960	0.000
	Female	726 (303)	807 (176)						
Speed (km·h^−1^)	Male	11.37 (0.80)	11.55(0.40)	0.219	0.028	<0.001	0.416	0.837	0.001
	Female	10.26 (0.82)	10.51(0.48)						
VO_2max_ (mL·kg·min^−1^)	Male	47.21 (4.49)	45.70(2.34)	0.129	0.043	<0.001	0.417	0.973	0.001
	Female	41.29 (4.35)	39.85(2.73)						
VO_2max_ (L·min^−1^)	Male	2.57 (0.41)	2.92 (0.34)	0.027	0.089	<0.001	0.445	0.126	0.044
	Female	2.12 (0.35)	2.19 (0.18)						

ES: effect size.

**Table 5:** Results obtained in the lateral movement, acceleration and reaction time tests.

		Under14	Under16	Group Effect (*p* Value)	ES	Gender Effect (*p* Value)	ES	Group x Gender (*p* Value)	ES
Dominant lateral movement (s)	Male	2.23 (0.09)	2.14 (0.13)	0.045	0.071	0.037	0.080	0.113	0.047
	Female	2.28 (0.14)	2.23 (0.12)						
Non-dominant lateral movement (s)	Male	2.31 (0.14)	2.25 (0.13)	0.806	0.001	0.030	0.086	0.502	0.009
	Female	2.34 (0.14)	2.39 (0.12)						
Dominant lateral acceleration (m·s^−2^)	Male	1.42 (0.18)	1.39 (0.26)	0.666	0.004	0.014	0.156	0.657	0.004
	Female	1.53 (0.40)	1.51 (0.21)						
Non-dominant lateral acceleration (m·s^−2^)	Male	1.44 (0.32)	1.55 (0.17)	0.851	0.003	0.339	0.017	0.147	0.039
	Female	1.61 (0.26)	1.53 (0.29)						
Dominant side reaction time (s)	Male	0.78 (0.21)	0.69 (0.27)	0.116	0.034	0.237	0.025	0.885	0.000
	Female	0.72 (0.33)	0.70 (0.26)						
Non-dominant side reaction time (s)	Male	0.87 (0.35)	0.76 (0.19)	0.857	0.001	0.021	0.103	0.298	0.025
	Female	0.75 (0.25)	0.87 (0.31)						

ES: effect size.

## Data Availability

Not applicable.
